# Treatment and monitoring of Philadelphia chromosome-positive leukemia patients: recent advances and remaining challenges

**DOI:** 10.1186/s13045-019-0729-2

**Published:** 2019-04-23

**Authors:** Simona Soverini, Renato Bassan, Thomas Lion

**Affiliations:** 1 Hematology/Oncology ‘L. e A. Seràgnoli’, Department of Experimental, Diagnostic and Specialty Medicine, University of Bologna, Via Massarenti 9, 40138 Bologna, Italy; 2 Division of Hematology, Ospedale dell’Angelo, Mestre, Venice, Italy; 3 Children’s Cancer Research Institute (CCRI) and Medical University of Vienna, Vienna, Austria

**Keywords:** Chronic myeloid leukemia, Acute lymphoblastic leukemia, BCR-ABL1, Minimal residual disease, Next-generation sequencing, Tyrosine kinase inhibitors, Chemotherapy, Allogeneic stem cell transplantation

## Abstract

The Philadelphia (Ph) chromosome, resulting from the t(9;22)(q34;q11) translocation, can be found in chronic myeloid leukemia (CML) as well as in a subset of acute lymphoblastic leukemias (ALL). The deregulated BCR-ABL1 tyrosine kinase encoded by the fusion gene resulting from the translocation is considered the pathogenetic driver and can be therapeutically targeted. In both CML and Ph-positive (Ph+) ALL, tyrosine kinase inhibitors (TKIs) have significantly improved outcomes. In the TKI era, testing for BCR-ABL1 transcript levels by real-time quantitative polymerase chain reaction (RQ-PCR) has become the gold standard to monitor patient response, anticipate relapse, and guide therapeutic decisions. In CML, key molecular response milestones have been defined that draw the ideal trajectory towards optimal long-term outcomes. Treatment discontinuation (treatment-free remission, TFR) has proven feasible in a proportion of patients, and clinical efforts are now focused on how to increase this proportion and how to best select TFR candidates. In Ph+ ALL, results of trials with second- and third-generation TKIs are challenging the role of intensive chemotherapy and even that of allogeneic stem cell transplantation. Additional weapons are offered by the recently introduced monoclonal antibodies. In patients harboring mutations in the BCR-ABL1 kinase domain, prompt therapeutic reassessment and individualization based on mutation status are important to regain response and prevent disease progression. Next-generation sequencing is likely to become a precious tool for mutation testing because of the greater sensitivity and the possibility to discriminate between compound and polyclonal mutations. In this review, we discuss the latest advances in treatment and monitoring of CML and Ph+ ALL and the issues that still need to be addressed to make the best use of the therapeutic armamentarium and molecular testing technologies currently at our disposal.

## Background

Chronic myeloid leukemia (CML) and a subset of acute lymphoblastic leukemias (ALL) are collectively termed “Philadelphia chromosome-positive (Ph+)” leukemias because they share a common pathogenetic lesion, the Philadelphia chromosome, resulting from the t(9;22)(q34;q11) translocation [1, 2]. On the Ph chromosome, a BCR-ABL1 fusion gene is formed that encodes a tyrosine kinase whose deregulated activity may be therapeutically targeted (reviewed in [3]). Since 2003, the incorporation of tyrosine kinase inhibitors (TKIs) in the front-line treatment protocols has significantly improved the prognosis of both diseases and has shifted treatment endpoints from hematologic and cytogenetic responses to molecular responses (MR). CML and Ph+ ALL are otherwise profoundly different in terms of disease biology and clinical course. Approximately 95% of CML patients present in the chronic phase (CP) that exhibits a relatively indolent course and is generally very well controlled by TKIs. CP CML patients who achieve an optimal response have a life expectancy comparable to that of the general population [4], and the possibility to discontinue TKI treatment (“treatment-free remission,” TFR) has recently been explored in several clinical trials [5–27]. Patients who present in (2–5%) or progress to accelerated phase (AP) or blast crisis (BC) (2–7%) have poorer outcomes [28, 29]. Patients in AP and BC display a high degree of genetic instability, leading to the accumulation of TKI-resistant point mutations in the BCR-ABL1 kinase domain (KD) [3]. Moreover, additional genetic and cytogenetic abnormalities reducing the addiction of Ph+ cells to BCR-ABL1 are observed, thereby limiting TKI efficacy [3]. Five different TKIs (imatinib, dasatinib, nilotinib, bosutinib, ponatinib) are approved for CML patients, permitting to tailor therapy at diagnosis and dynamically adapt thereafter on the basis of disease phase, individual risk assessment, treatment endpoints, comorbidities, response levels, and, in case of treatment failure, presence of TKI-resistant mutations in the BCR-ABL1 KD [30–32]. In Ph+ ALL, the introduction of TKIs has enabled the achievement of very high rates of complete hematologic response (CHR) and has significantly improved disease-free survival (DFS) and overall survival (OS) [33]. Nevertheless, several open issues remain to be addressed on the path to treatment optimization, and development of resistance is still a major concern. The degree of genetic instability in Ph+ ALL resembles that of BC CML and, in both instances, fosters rapid acquisition of BCR-ABL1 KD mutations that may lead to TKI-resistant relapse.

Here, we discuss why MR monitoring and BCR-ABL1 KD mutation screening have become essential for the successful management of Ph+ leukemia patients and how molecular tools are evolving. We also review the therapeutic landscape of Ph+ ALL and the challenges that still need to be addressed in order to improve patient outcomes.

## Main text

### What is new in minimal residual disease (MRD) monitoring of CML: early molecular response and BCR-ABL1 transcript kinetics

It is well established that the achievement of given MR milestones at defined timepoints during therapy, as assessed by real-time quantitative reverse transcription-polymerase chain reaction (RQ-PCR) draws the ideal trajectory towards optimal outcomes [30, 31] and, possibly, TFR [34]. The first important checkpoint for response assessment is as early as after 3 months of therapy, when the achievement of a BCR-ABL1 transcript level < 10% on the International Scale (IS) [35] defines the so-called Early Molecular Response (EMR). Several retrospective studies have shown that EMR at 3 months predicts for significantly better long-term outcomes (event-free survival [EFS], progression-free survival [PFS], and overall survival [OS]) both in patients treated with imatinib [36, 37] and in patients treated with second-generation TKIs [38–42]. However, the European LeukemiaNet (ELN) and the more recent European Society of Medical Oncology (ESMO) recommendations have not considered a single BCR-ABL1 measurement as sufficient to trigger a change of therapy, by defining BCR-ABL1 ≥ 10% at 3 months as a “warning” but not as a “failure” of therapy [30, 31]. This recommendation is supported by the observation that a sizeable proportion of patients may still achieve an optimal response later on, and there are no studies proving that an early switch would result in an improved outcome. More recently, it has been recognized that a trend in BCR-ABL1 transcript reduction is indeed much more informative than a single value: the kinetics of BCR-ABL1 transcripts during the first 3 months has thus been proposed as a more reliable indicator of the ensuing molecular response and outcome. Branford et al. introduced the concept of “halving time of BCR-ABL1 transcripts” and showed that in patients on first-line imatinib treatment who fail to achieve the threshold of 10% at 3 months, a halving time of less than 76 days is associated with significantly superior outcomes [43]. Similar data were also published by the German Study Group, which showed that the reduction of BCR-ABL1 transcripts within the first 3 months by half a log or more also predicts for better PFS and OS [44]. This reflects the fact that the actual baseline level of BCR-ABL1 transcripts varies greatly among individual patients, and the presence of similar levels at 3 months can therefore either mirror a substantial decline of BCR-ABL1 transcripts or only a minimal (if any) reduction of the pre-TKI value. Two more recent studies have reported similar observations in patients on first-line treatment with second-generation TKIs: a Japanese study indicated that patients with a halving time of 14 days or less had a higher likelihood to achieve major molecular response (MMR; BCR-ABL1 ≤ 0.1%^IS^) and MR^4^ (4-log reduction in transcript level on the IS) on dasatinib therapy [45], and a Nordic study from Finland, Sweden, and Norway suggested that a greater than 1-fold decline of BCR-ABL1 transcripts after 1 month of therapy with imatinib, nilotinib, or dasatinib is associated with better responses at 3 months and significantly higher rates of MMR [46]. To assess the early response to TKI treatment by determining the kinetics of BCR-ABL1 transcripts, a minimum of two, but preferentially more consecutive measurements would be required, e.g., at baseline and subsequently at monthly intervals during the first 3 months. However, analysis of a much larger series of patients will be necessary to establish harmonized, clinically applicable values for halving time or fold reduction before considering the routine application of BCR-ABL1 transcript kinetics measurement. From a technical standpoint, some changes in procedures will also be needed. First, whereas the expression of results on the IS currently requires the use of a standardized baseline [35], RQ-PCR assessment of patient-specific BCR-ABL1 baseline levels will have to be implemented to permit evaluation of transcript kinetics. Second, monthly sampling of peripheral blood for MR assessment during the first 3 months will have to be scheduled. Third, switch to a control gene other than ABL1—that is presently the most widely used one—will be necessary, at least during the first months of therapy. This is because the PCR primers used for amplifying the ABL1 control gene also amplify the target sequence from the BCR-ABL1 fusion transcript, leading to an underestimation of the actual BCR-ABL1/ABL1 ratio at early timepoints, when the BCR-ABL1 transcript levels are still high. The ideal candidate gene would be beta-glucuronidase (GUSB) [35], recommended years ago together with ABL1 after a thorough experimental evaluation of a series of control genes. Alternatively, a parallel assessment of both ABL1 and GUSB should be considered. It is thus premature to expect the incorporation of early BCR-ABL1 transcript kinetics into treatment recommendations. Nevertheless, it would be advisable to start employing monthly monitoring of MR during the first 3 months of TKI therapy in order to accumulate data that may serve as a basis for future optimization of diagnostics and algorithms of treatment decision.

### The latest frontiers in CML treatment: deep molecular response and treatment-free remission

Beyond EMR, two additional molecular response milestones have been defined. Major molecular response (MMR; BCR-ABL1 ≤ 0.1%^IS^) was introduced at the times of the IRIS study (International Randomized Study of Interferon and STI-571, the phase 3 study that led to imatinib registration in the first line) [47] and has long been considered “the safe haven” to aim for. However, with longer follow-up of patients on imatinib and the availability of more potent, second-generation TKIs, it became evident that deeper responses could be achieved in increasing proportions of patients. In recent years, international efforts aimed at optimizing methodologies and providing guidelines for reliable, standardized assessment and definition of deep responses [48] have been instrumental to explore the clinical significance of response levels below MMR. Deep molecular response (DMR) is defined as BCR-ABL1 ≤ 0.01%^IS^. DMR can be further stratified into MR^4^, MR^4.5^, or MR^5^ depending on the extent of log reduction in BCR-ABL1 transcript levels from the standardized baseline of the IS (4, 4.5, or 5 logs, respectively) and on the control gene copy number [49]. Achievement of DMR is per se an important prognostic factor for long-term clinical outcome [50–52], but it is also thought to be the gateway to TFR and “functional cure” of CML.

The earlier the MR milestones are achieved, the greater the chance of reaching stable DMR, which is the prerequisite for TFR. A study by Branford et al. has shown that the cumulative incidence of (stable) MR^4.5^ after 8 years of imatinib therapy correlates significantly with lower BCR-ABL1 values at 3 months and faster achievement of MMR [53].

A number of clinical studies addressing TKI cessation in patients with stable DMR have been conducted over the past years or are currently ongoing (Table 1) [5–27]. The inclusion criteria varied, requiring a minimum TKI treatment duration of 2–3 years and a depth of molecular response at the level of MR^4^ or MR^4.5^ in most studies. The minimum duration of this MR prior to TKI discontinuation was 1–2 years in most instances, and the relapse-free survival ranged between 40 and 60%, but the observation time in some studies is still relatively short. Recommendations on treatment discontinuation have not yet been formulated by the ELN, but guidelines from the National Comprehensive Cancer Network (NCCN) [32] are available, and a series of expert reviews on this topic have also been recently published [34, 54–57]. While there is general agreement regarding patients with non-high Sokal score who display a typical BCR-ABL1 transcript (b2a2 or b3a2) and do not have warning signs or failure responses in their clinical history as the ideal candidates for TFR [32, 34, 54–57], there is limited consensus on the requirements for TKI treatment duration or depth and stability of DMR. The general perception regarding treatment duration is *the longer the better*, but with respect to the depth of molecular response, it is still a matter of investigation whether the deeper the better. The choice of a suitable reference laboratory is essential for reliable selection and appropriate surveillance of patients who are candidates for TFR. An appropriate laboratory should (i) have a regularly validated conversion factor for the expression of results on the IS, (ii) be able to reliably measure MR^4.5^ and MR^5^ in the majority of samples, (iii) be able to perform RQ-PCR tests every 4–6 weeks, and (iv) ensure rapid turn-around time for reporting results (within 4 weeks) [57]. Although RQ-PCR remains the gold standard for MR assessment before and during discontinuation, a series of ongoing studies are investigating whether a digital PCR-based approach might better stratify deep responders and whether this may contribute to increased TFR rates.

**Table 1:** Summary of discontinuation studies published as full papers or in abstract form, with MR levels required for inclusion and for the definition of relapse

Study (ref)	No. of patients	Treatment before discontinuation	Requirements to stop therapy	Definition of relapse	TFR rate
STIM 1 [5]	100	Imatinib (1st line or after IFN) for 3 years	CMR (undetectable transcript) for ≥ 2 years	Loss of CMR or ≥ 1-log increase in BCR-ABL	39% @ 77 months
STIM 2 [6]	124	Imatinib (1st line or after IFN) for ≥ 3 years	As for STIM	As for STIM	61% @ 12 months
TWISTER [7]	40	Imatinib (1st line or after IFN) for ≥ 3 years	Undetectable transcript for ≥ 2 years	Loss of MMR or confirmed loss of MR^4.5^	42.7% @ 24 months
A-STIM [8]	80	Imatinib (1st line) for ≥ 3 years	As for STIM; occasional positive samples eligible	Loss of MMR	61% @ 36 months
KIDS [9]	48	Imatinib (1st line or after IFN)	Undetectable transcript for ≥ 2 years	Loss of MMR	58.5% @ 24 months
JALSG-STIM213 [10]	77	Imatinib (1st line or after IFN)	MR^4^ for ≥ 24 months (4 PCR)	Loss of MMR	67.6% @ 12 months
ISAV [11]	112	Imatinib (1st line or after IFN)	Undetectable transcript for ≥ 18 months (3 PCRs)	Loss of MMR	52% @ 22 months
EUROSKI [12]	758	Imatinib (1st line or after IFN), dasatinib, nilotinib	MR^4^ for ≥ 1 year; TKI for ≥ 3 years	Loss of MMR	50% @ 24 months
STOP 2G-TKI [13]	60	Nilotinib or dasatinib (2nd line)	Undetectable transcript for ≥ 2 years	Loss of MMR	63.3% @ 12 months
DADI [14]	63	Dasatinib (2nd line)	MR^4^ for ≥ 1 year (4 PCR)	Loss of MR^4^	44.4% @ 36 months
ENEST freedom [15, 16]	190	Nilotinib (1st line)	MR^4.5^ for ≥ 2 years	Loss of MMR	48.9% @ 96 weeks
ENESTop [17]	126	Nilotinib (2nd line, after imatinib)	MR^4.5^ for ≥ 2 years	Confirmed loss of MR^4.0^or any loss of MMR	53.2% @ 96 weeks
DESTINY [18]	174	Imatinib, dasatinib, nilotinib (50% de-escalation for 12 months, then stop)	At least stable MMR for 12 months (3 PCR) and stable response under half standard dose for 12 months	Loss of MMR	73% in pts. with stable MR^4^; 41% in pts with stable MMR
D-STOP [19]	65	Dasatinib as consolidation for 2 years	MR^4^ for ≥ 2 years	Loss of confirmed MR^4^	62.9% @ 12 months
DASFREE [20]	84	Dasatinib (1st or subsequent line)	MR^4.5^ for ≥ 1 year	Loss of MMR	48% @ 18 months
TRAD [21]	131	Dasatinib rechallenge and discontinuation after imatinib discontinuation (second-stop)	MR^4.5^ for ≥ 2 years	Loss of MR^4^ on 2 consecutive occasions or loss of MMR on 1 occasion	21.5% @ 6 months
NILSt [22]	112	Nilotinib (1st line or after imatinib)	MR^4.5^ for 2 years	Loss of MR^4.5^	61% @ 12 months
LAST [23]	173	Imatinib, dasatinib, nilotinib, or bosutinib	MR^4^ for ≥ 2 years	Loss of MMR	60% @ 12 months
STAT2 [24]	96	Nilotinib as consolidation for 2 years	MR^4.5^ for 2 years	Confirmed loss of MR^4.5^	67.9% @ 12 months
ENESTpath [25]	619	Nilotinib (2nd line, after imatinib)	Randomized MR^4.5^ for ≥ 1 year vs ≥ 2 year	Confirmed loss of MR^4^ or any loss of MMR	In progress
ENESTGoal [26]	59	Nilotinib (2nd line, after imatinib)	MR^4.5^ for ≥ 1 year	Confirmed loss of MR^4^ or any loss of MMR	In progress
CML V [27] (TIGER)	717	Randomized nilotinib vs nilotinib + pegIFN (1st line)	MR^4^ for ≥ 1 year	Loss of MMR	In progress

*Abbreviations*: *IFN* interferon, *pegIFN* pegylated IFN, *PCR* polymerase chain reaction, *CMR* complete molecular response, *MMR* major molecular response

### Ph+ ALL: optimization of remission induction regimens

Besides CML, the Ph translocation can be detected in acute leukemia patients. While Ph+ acute myeloid leukemia is very rare, in adults Ph+ ALL is the most frequent ALL subtype expressing a recurrent cytogenetic/genetic abnormality. At present, clinical research questions are different in Ph+ ALL as compared to CML. The primary goal of therapy is to induce a CHR. Several clinical studies conducted over the past 20 years with TKIs administered within different schedules and combinations with chemotherapy have led to a major therapeutic advancement, with CHR rates between 90 and 100% (Table 1) [58–83]. It soon became clear that the risk of resistant disease was sensibly reduced, if not totally abolished, compared to historical results of the pre-TKI era. Today, for patients who are not enrolled in a clinical trial, we may have access to more than one effective TKI, although in many countries only imatinib is licensed for use in untreated patients, while second-generation TKIs and ponatinib are reserved for patients who are resistant/intolerant to either imatinib or dasatinib/nilotinib or carry the T315I KD mutation. The main open questions about TKI-based induction therapy are as follows: is associated chemotherapy necessary? And if so, in which form? And then, if the selection of treatment is possible outside a clinical trial and regardless of local regulations, is there a better TKI to use? The issue of associated chemotherapy requires a careful analysis of clinical trial results, especially in older patients who are at greater risk of early death by infections and hemorrhage after intensive chemotherapy. This consideration led the Gruppo Italiano Malattie Ematologiche dell’Adulto (GIMEMA) group to test whether a chemotherapy-free schedule, with TKI monotherapy plus corticosteroids, could be as effective, but less toxic than a combined intensive induction treatment. The first trial, conducted in the most critical setting of elderly Ph+ ALL, reached a notable 100% CHR rate in a group of patients displaying a median age of 69 years [79]. A comparable, small randomized trial performed by the German Multicenter Study Group for Adult ALL (GMALL) [61] provided similar results (no induction deaths) with a low incidence of resistance (4%). Subsequent GIMEMA studies confirmed the value of monotherapy with either imatinib, or dasatinib, or imatinib alternating with nilotinib, or ponatinib (CHR 95–100%) [58, 80–83], with only occasional induction of resistance or occurrence of death and a good toxicity profile in all age groups, including unfit patients. Other studies explored combinations of TKI plus low-intensity chemotherapy, again with favorable results and very low to absent induction of resistance or deaths (each < 3%) [68, 76–79]. Compared to intensive chemotherapy schedules (Table 2) [59–75], both low-dose and no chemotherapy approaches yielded superimposable or even slightly higher CR rates, because of the lower or absent early mortality, in contrast to higher numbers after intensive regimens (Table 2). Of great interest was the randomized GRAAPH-2005 trial, which tested an attenuated imatinib-based induction (plus vincristine/dexamethasone) vs. the aggressive Hyper-CVAD (cyclophosphamide, vincristine, adriamycin, dexamethasone) regimen [68]. The CHR results were clearly in favor of the non-intensive arm, due to the significant reduction of early toxic deaths (1.7% vs. 6.7%, *P* = 0.01); moreover, long-term results were comparable between study arms, with no detrimental effect on survival with lower intensity induction. From the results of these studies, it may be argued that non-intensive induction schedules are at least as effective as intensive chemotherapy regimens for CR induction, with the benefit of a safer toxicity profile. On the other hand, there is no evidence as yet about the long-term results of fully non-intensive schedules, because a variably intensive chemotherapy-based post-remission consolidation was included in almost all attenuated or no chemotherapy induction studies. A related point of concern is the management of the patients who exhibit high-risk features and/or are unable to undergo a subsequent allotransplantation. For these cases, intensive chemotherapy/TKI associations may still be recommended. The question regarding which TKI to choose is a difficult one, because of the comparably high CHR rates reported for all regimens, including simple imatinib monotherapy. However, many of the patients exhibiting TKI resistance in these studies were found to have BCR-ABL1 KD mutations. When first- and second-generation TKIs were used, the most frequent mutation was the T315I [84]—a piece of information which may support a personalized TKI choice (see the paragraph below on predictive “heatmaps”). In therapy-naïve patients, the issue of resistance related to mutations, including the T315I mutation, would be less of a concern with ponatinib. In addition, we should consider the effects of induction and early consolidation therapy with different TKIs and chemotherapy combinations on the next most important step after CHR: the achievement of a major or complete (CMR) molecular response, i.e., an MRD-negative CHR.

**Table 2:** CR induction results from representative series of adult/elderly Ph+ ALL, by type of TKI and associated induction drugs. Reports from randomized trials are marked with an asterisk (*)

Study (author, ref.)	No. of patients	Patient median age, years (range)^1,2^	TKI	Associated drug regimen	Induction results (%)		
					CR	NR	ED
TKI + intensive chemotherapy							
Yanada (2006) [59]	80	48 (15–63)	IM	JALSG ALL202	96.2	1.3	2.5
Wassmann (2006) [60]	45	41 (19–63)	IM	GMALL	96	2	–
Ottmann (2007) [61]*	27	68 (58–78)^2^	IM	GMALL (intensive arm)	85	7	8
De Labarthe (2007) [62]	45	(16–59)	IM	GRAAPH-2003	93.5	–	6.5
Pfeifer (2010) [63]	284	43 (17–65)	IM	GMALL	87	4.2	8.8
Bassan (2011) [64]	59	45 (20–66)	IM	NILG 09/00	92	4	4
Ribera (2012) [65]	59	40 (15–62)	IM	PETHEMA	95.5	1.5	3
Thyagu (2012) [66]	32	46 (18–60)	IM	DFCI modified	93.7	6.3	–
Fielding (2014) [67]	89	42 (16–64)	IM	UKALL XII/ECOG 2993	92	1	7
Chalandon (2015) [68]*	133	45 (18–59)	IM	Hyper-CVAD (intensive arm)	91	2.2	6.7
Daver (2015) [69]	45	51 (17–84)	IM	Hyper-CVAD	93	3.5	3.5
Lim (2015) [70]	87	41 (16–71)	IM	Multiagent	94	–	6
Wang (2018) [71]	145	37 (14–65)	IM	CODP	94	4	2
Ravandi (2015) [72]	72	55 (21–80)	DAS	Hyper-CVAD	96	–	4
Kim (2015) [73]	90	47 (17–71)	NIL	Multiagent intensive	91	–	9
Ravandi (2016) [74]	94	44 (20–60)	DAS	Hyper-CVAD	88	9	2^3^
Jabbour (2018) [75]	76	47 (39–71)	PON	Hyper-CVAD	100^4^	–	–
TKI+ non-intensive chemotherapy							
Bassan (2010) [64]	67^5^	–	IM	Low intensity	100	–	–
Chalandon (2015) [68]*	135	49 (18–59)	IM	Low intensity (non-intensive arm)	98.5^6^	0.7	0.7
Rousselot (2016) [76]	71	69 (59–83)^2^	DAS	Low intensity	96	1	3
Chalandon (2018) [77]	60	47 (18–59)	NIL	Low intensity (non-intensive arm)	98.3	–	1.7
Ottmann (2018) [78]	72	65 (55–85)^2^	NIL	Low intensity	94.4	2.8	2.8
TKI without chemotherapy (prednisone only)							
Vignetti (2007) [79]	29	69 (61–83)^2^	IM	Prednisone	100	–	–
Ottmann (2007) [61]*	28	66 (54–79)^2^	IM	–	96^7^	4	–
Foà (2011) [80]	53	54 (24–76)	DAS	Prednisone	100	–	–
Papayannidis (2013) [81]	36	66 (28–84)	IM/NIL^8^	Prednisone	94	6	–
Chiaretti (2015) [58]	60	42 (18–59)	DAS	Prednisone	96.6	3.4	–
Chiaretti (2016) [82]	49	46 (17–59)	IM	Prednisone	96	–	4
Martinelli (2017) [83]	42	68 (27–85)^2^	PON	Prednisone	95.2	NA	NA

^1^Including elderly (> 55 years) and/or frail patients only

^2^Two patients not in CR by week 6

^3^All 65 patients with active disease at enrolment

^4^From modified NILG 09/00 protocol (low-intensity induction: no *L*-asparaginase, 50% idarubicin reduction; data on file)

^5^Randomized phase 3 trial: higher CR rate in the non-intensive arm (*P* = 0.006) due to lower ED rate (*P* = 0.010)

^6^*P* = 0.001 for CR rate vs intensive chemotherapy arm

^7^Alternating schedule (every 6 weeks)

^8^Two patients entering CR by day 57, losing response by day 85

*Abbreviations*: *IM* imatinib, *DAS* dasatinib, *NIL* nilotinib, *PON* ponatinib, *CR* complete remission, *NR* non-responsive, *ED* early death, *NA* not available

### The significance of molecular remission in Ph + ALL

Once a CHR is achieved, optimal (and durable) MRD response is the next major clinical goal, as well as an important determinant of long-term survival [85]. Almost 20 years of MR monitoring in CML have set the stage for routine use of RQ-PCR for response monitoring in Ph+ ALL as well. The increasing importance of MRD assessment in Ph+ ALL has fostered cooperative efforts aiming for standardization of molecular monitoring. The EURO-MRD Consortium including diagnostic centers from 15 European countries, USA, Brazil, and Singapore is actively pursuing the standardization of methodologies with the aim to reduce inter-laboratory variability, minimize the rate of false positive and false negative results, increase sensitivity, optimize reagents and procedures, establish a common terminology, and standardize interpretation and reporting of results. Of note, consensus guidelines for MRD assessment in Ph+ ALL by RQ-PCR have just recently been established [86].

The comparative analysis of induction and post-induction MRD results obtained with different TKIs (and chemotherapy) before transplantation allows to evaluate the efficacy of different treatment schedules, also within the different risk subsets, and helps establish priorities for clinical studies (Table 3) [63, 68, 69, 72, 73, 75–79, 81, 83, 87]. Before analyzing these data, it must be underlined how chemotherapy alone could transiently induce an MRD-negative status. Response to selected chemotherapy agents (anthracyclines) and synergistic effects of TKI-chemotherapy combinations were reported [85, 87–89]. The GET-LALA reported the BCR-ABL1 status of 63 patients after two intensive chemotherapy courses: 24 were MRD-negative (38%) [89]. A GIMEMA study reported a transcript reduction > 3 logs in 28/42 CHR patients (67%) after anthracycline-rich induction and early consolidation therapy [90]. These facts point to the usefulness of associated chemotherapy, at least in selected patients who have no access to innovative drugs and/or display clinical or molecular TKI resistance. Post-induction MRD results from TKI-based studies were quite variable, depending on TKI type and associated chemotherapy (Table 3). First, imatinib or dasatinib with or without chemotherapy yielded favorable early MRD responses (CMR, MMR) in the 20% range [58, 63, 68, 72, 79], with no differences between intensive and non-intensive chemotherapy, as shown by the phase III GRAALL trial [67]. Nilotinib appeared to perform better, although no monotherapy study is available. In combination with chemotherapy of variable intensity, early CMR rates with nilotinib were close to 60%, and MMR rates close to 80% [73, 77, 78]. The best MRD results were reported with ponatinib, with CMR rates of 60–80% (the higher figure in association with intensive chemotherapy) [75, 83], and an outstanding MMR rate of 97% in one study [85]. These results depict a complex pattern of MRD response, affected both by TKI type and by associated chemotherapy, and offer a clue to develop increasingly effective induction schedules—including a shift to the more active compounds if less than MMR is achieved on first- and second-generation TKIs and/or if BCR-ABL1 KD mutations or other adverse genomic markers are detected early on. Whatever the CMR/MMR findings and the efficacy of salvage therapy in poor MRD responders, consolidated CHR patients face the next most important step towards achieving a “cure,” that is, an allogeneic stem cell transplantation (SCT).

**Table 3:** Post-induction and pre-transplantation MRD responses in representative, selected series of adult/elderly Ph+ ALL, by type of TKI and associated induction chemotherapy. The single randomized trial is indicated by an asterisk (*)

Study (author, ref.)	TKI-based therapy		No. of patients with CHR	Post-induction MRD (%)^1^	
				CMR	MMR
	TKI	Chemotherapy			
Daver (2015) [69]	IM	Intensive (Hyper-CVAD)	51	45 (12 weeks)	38 (median 10 weeks)
Pfeifer (2010) [63]		Intensive (GMALL)	247	12.5–33 (consolidation 1)^2^	–
Chalandon (2015) [68]		Intensive (GRAALL)*	121	9.5 (cycle 1) 28.6 (cycle 2)	43.1 (cycle 1) 66.1 (cycle 2)
		Non-intensive (GRAALL)*	133	9.9 (cycle 1) 22.6 (cycle 2)	45.5 (cycle 1) 64.5 (cycle 2)
Vignetti (2007) [79]		None (GIMEMA)^3^	29	14	–
Ravandi (2015) [72]	DAS	Intensive (Hyper-CVAD)	69	65 (median 4 weeks)	28 (median 4 weeks)
Rousselot (2016) [76]		Non-intensive (EWALL)	67	20 (cycle 1) 24 (cycle 2)	60 (cycle 1) 65 (cycle 2)
Chiaretti (2015) [58]		None (GIMEMA)^3^	58	18.6	–
Kim (2015) [73]	NIL	Intensive	82	56 (at CHR)	79 (at CHR)
Ottmann (2018) [78]		Non-intensive (EWALL)	68	14 (cycle 1) 58 (consolidation 2)	41 (cycle 1) 86 (consolidation 2)
Chalandon (2018) [77]		Non-intensive (GRAALL)	60	–	80 (cycle 2) 93 (cycle 4)
Papayannidis (2013) [81]	IM/NIL	None (GIMEMA)^4^	34	35.4 (week 6) 46.6 (week 12)	–
Jabbour (2018) [75]	PON	Intensive (Hyper-CVAD)	76	83 (median 10 weeks)	97 (median 3 weeks)
Martinelli (2017) [83]		None (GIMEMA)^3^	38	60.6	–

^1^Non-standard definitions according to single studies: CMR, complete molecular response (BCR-ABL1 MRD < 0.01–0.001% or undetectable); MMR, major molecular response (BCR-ABL1 MRD < 0.1%); results after treatment course/week/time as indicated

^2^IM starting with induction Ib vs. Ia, respectively

^3^Plus systemic corticosteroids and intrathecal prophylaxis (methotrexate)

^4^Alternating schedule q6 weeks

*Abbreviations: IM* imatinib, *DAS* dasatinib, *NIL* nilotinib, *PON* ponatinib, *CHR* complete hematological response, *CMR* complete molecular response, *MMR* major molecular response

### The allogeneic SCT choice in Ph+ ALL

An allogeneic SCT has long been the only means to reach a cure in a sizeable fraction of Ph+ ALL patients [85, 91]. By improving CHR rate and duration, TKI-based treatments allowed to transplant more patients in the first CHR, from both related and unrelated HLA-matched donors. In the TKI era, an allogeneic SCT can be performed in 45–80% of CHR patients [91], representing a major contribution to an overall survival of 35–55% at 2–5 years, and up to 60–70% among allografted patients (Table 4) [58, 63–75, 82]. These data established the current standard treatment paradigm for adult Ph+ ALL, consisting of a TKI-based induction/consolidation followed by an allograft—however with a number of caveats. The limitations of allogeneic SCT typically concern elderly patients, many of whom cannot simply be transplanted. Moreover, inferior efficacy can be observed in carriers of the T315I mutation, CKND2A/2B deletions, or other genetic abnormalities and in patients transplanted in an MRD-positive status [92–97]. These patients are at high risk of post-transplantation relapse and may not convert to MRD negativity. For these reasons, all the patients require a careful post-transplantation MRD monitoring for a timely reinstitution of TKI therapy or other interventions [98, 99]. Patients older than 50–55 years display an increased risk of transplant-related mortality (TRM). The issue of TRM is crucial, with an incidence as high as 25% in large recent reference series like the GRAALL study [68] and others (Table 4). An update of two Northern Italy Leukemia Group (NILG) studies [94] indicated a TRM incidence of 20% and 33% in the two cohorts of MRD-negative and MRD-positive patients, respectively. A very recent update of the German study in 07/2003 on 239 allografted patients (median age 40 years) reported a 5-year survival of 59% with a TRM rate of 25% [100]. These facts led to consider a reduced-intensity conditioning (RIC) for SCT, which lowered the 1-year incidence of TRM from 36% with myeloablative conditioning to 13% (*P* = 0.001) while preserving the SCT efficacy in MRD-negative but not MRD-positive patients [101].

**Table 4:** Long-term results of TKI-based clinical trials for adult Ph+ (patients in complete hematologic remission), with emphasis on allogeneic stem cell transplantation (SCT)

Study (author, ref.)	No. of patients	TKI-based therapy	Treatment outcome (%)						
			General	SCT			No SCT		
				No.	Outcome	TRM	No.	Outcome	*P* (vs. SCT)
Pfeifer (2010) [63]	247	IM/CT	40–50 (OS, 4 years)	180	57 (OS, 3 years) 72 (DFS)	21–26	–	14 (OS, 3 years)	NR
Bassan (2010) [64]	53	IM/CT	38 (OS, 5 years) 39 (DFS)	34	46 (DFS, 5 years)	17	19	30 (OS, 2 years)	0.019 (DFS)
Ribera (2012) [65]	56	IM/CT	37–63 (EFS, 2 years)	32	NR	31	24	NR	NR
Thyagu (2012) [66]	30	IM/CT	53 (OS, 3 years) 50 (EFS)	16	56 (OS, 3 years) 70 (EFS)	37.5	14	50 (OS, 3 years) 45 (EFS, 3 years)	0.34 (OS) 0.51 (DFS)
Fielding (2014) [67]	161	IM/CT	38 (OS, 4 years) 50 (DFS) 33 (EFS)	93	52 (OS, 4 years) 72 (DFS) 49 (EFS)	NR	44	19 (OS, 4 years) 14 (DFS) 14 (EFS)	NR
Chalandon (2015) [68]	254	IM/CT	45.6 (OS, 5 years) 37.1 (EFS)	148	56.7 (OS, 5 years) 48.3 (DFS)	25.8	106	35 (OS, 5 years) 28 (DFS)	0.02 (OS) 0.03 (DFS)
Daver (2015) [69]	39	IM/CT	43 (OS. 5 years) 43 (DFS)	16	63 (DFS, 5 years)	NR	23	43 (DFS, 5 years)	0.52 (DFS)
Lim (2015) [70]	82	IM/CT	39(OS, 5 years) 33 (DFS)	56	53 (OS, 5 years) 43 (DFS)	30	26	NR	NR
Chiaretti (2016) [72]	47	IM/CT	48.8 (OS, 5 years) 45.5 (DFS, 5 years)	23	NR	13	24	NR	0.03 (OS)
Wang (2018) [71]	136	IM/CT	69.2 (OS, 4 years) 61 (DFS)	77	82.6 (OS, 4 years) 71.3 (DFS)	10	56	45.6 (OS-4 years) 43.9 (DFS)	< 0.001 (OS, DFS)
Chiaretti (2015) [58]	60	DAS/CT	58 (OS, 3 years) 49 (DFS, 3 years)	NR	NR	NR	NR	NR	NR
Ravandi (2016) [74]	83	DAS/CT	69 (OS, 3 years) 62 (DFS) 55 (EFS)	41	76 (DFS, 3 years)	0	53*	56 (OS, 3 years) 51 (DFS)	0.037 (OS) 0.038 (DFS)
Kim (2015) [73]	82	NIL/CT	72 (OS, 2 years)	57	78 (DFS, 2 years)	19	25	49 (DFS, 2 years)	0.045 (DFS)
Ottmann (2018) [78]	68	NIL/CT	47 (OS, 4 years) 42 (EFS)	24	61 (OS, 4 years)	25	44	39 (OS, 4 years)	NS
Jabbour (2018) [75]	76	PON/CT	71 (OS, 5 years) 83 (DFS, 3 years) 67 (EFS)	15	70 (OS, 3 years)	20	61	87 (OS, 3 years)	0.32 (OS)

*Including eight no-protocol SCT patients

*Abbreviations: OS* overall survival; *DFS* disease-free survival; *EFS* event-free survival, shown is a long-term estimate at 3+ years (length of follow-up); *CT* chemotherapy; *TRM* transplant-related mortality; *NR* not reported; *NS* not significant

### Opening to “no alloSCT approaches” in Ph+ ALL

The significant morbidity and mortality associated with allogeneic SCT prompted the search for a different therapeutic approach, at least in CHR patients with a better risk profile. An autologous SCT followed by long-term TKI maintenance was demonstrated to be relatively effective for MRD-negative patients in some trials and in a retrospective analysis of the European Society for Blood and Marrow Transplantation (EBMT) [64, 68, 102]. However, although TRM was significantly reduced with autologous compared to allogeneic SCT (2% vs. 20%, *P* = 0.0001), the advantage was offset by the higher relapse rate (47% vs. 28% and 19% with related and unrelated donor SCT, respectively, *P* = 0.0002) [102]. Perhaps more interesting is a totally transplant-free strategy. Many of the trials summarized in Table 4 reported survival rates around 30–50% at 2–5 years in non-SCT patients [66, 68, 69, 71, 73–75, 78], with minimal or statistically non-significant differences as compared to SCT-treated patients [59, 69, 73, 75, 103, 104]. The most relevant findings were obtained from studies with dasatinib/nilotinib/ponatinib associated with chemotherapy [73, 75, 78]. The most striking example was the ponatinib/chemotherapy phase 2 trial from the M.D. Anderson Hospital [75]. In that study, very recently updated, the 3-year overall survival was 70% for allografted patients (*n* = 15, TRM 20%) compared to 87% for the 61 patients who continued on study drugs after the achievement of a major/complete MRD response. The M.D. Anderson team had previously demonstrated that 62% of the CHR patients in CMR status at 3 months on imatinib/dasatinib-based programs remained disease-free at 4+ years [103]. A Chinese study reported an excellent 84% disease-free survival without allogeneic SCT in low-risk patients identified by a presenting leukocyte count < 30 × 10^9^/l and a good MRD response (≥ 3 logs) [71].

### New challenges and opportunities in Ph+ ALL treatment

Therapeutic progress rests on the availability of new powerful drugs and the design of prospective clinical trials that advance treatment strategies. Starting with imatinib, any subsequent new TKI or targeted agent, such as the antibody-drug conjugate inotuzumab ozogamicin and the bispecific antibody blinatumomab, were only partially effective when used in relapsed/refractory patients [105, 106], calling for an upfront evaluation of their exceptional properties, prior to the expansion of highly resistant subclones of the disease. The new first-line programs adopt TKI/immunotherapy or TKI/other targeted therapy combinations [107], with a preference for second/third generation TKIs and a progressive reduction or abolishment of systemic chemotherapy: ponatinib/blinatumomab (ClinicalTrials.gov Identifier: NCT03263572), dasatinib/blinatumomab (NCT02003222), dasatinib/ruxolitinib (NCT02494882), dasatinib/ibrutinib (NCT02815059), low-dose chemotherapy plus imatinib or ponatinib, phase 3 trial (NCT03589326), low-dose chemotherapy plus imatinib or ponatinib vs ponatinib/blinatumomab, 3-arm phase 3 trial (planned by the European Working Group on Adult ALL [EWALL]). Moreover, we can anticipate an increasing attention towards a risk-modeled allotransplantation strategy supported by an in-depth evaluation of the molecular mechanisms of resistance and MRD analysis [90, 108].

### Current and future approaches for BCR-ABL1 KD mutation screening in TKI-resistant patients

In both CML and Ph+ ALL, lack or loss of response to TKI therapy is frequently associated with the selection of point mutations in the BCR-ABL1 KD [109]. Almost a hundred of imatinib-resistant mutations scattered across the entire KD have been reported. By contrast, only a small number of mutations resistant to second-generation TKIs, which tend to be limited to critical contact residues (T315, Y253, E255, and F359 for nilotinib; T315I, V299, and F317 for dasatinib; T315, V299, and E255 for bosutinib), display clinical relevance [110]. In CML, mutations are more common in cases of acquired resistance as opposed to the presence of primary resistance. Moreover, the likelihood of detecting a mutation in patients who fail TKI treatment increases from CP to BC, ranging from approximately 30% to more than 70% [111]. In Ph+ ALL, mutations have been reported in almost 70% of imatinib-resistant patients and in almost 80% of patients who develop resistance to a second-generation TKI after imatinib failure [84]. In CML-BC and Ph+ ALL, the most frequent mutation is the T315I [94, 100] conferring resistance to imatinib and all second-generation TKIs. Currently, it may only be overcome by the third generation TKI ponatinib. Sequential treatment by different TKIs may favor the development of “compound” mutations (CMs; i.e., more than one mutation in the same BCR-ABL1 molecule, reflecting a specific leukemic subclone) [112]. The occurrence of compound mutations has been observed particularly in patients with CML-BC and Ph+ ALL, where genetic instability fostering the acquisition of further mutations is high, thus increasing the likelihood of subsequent TKI-resistant relapses. The great majority of CMs have been predicted to display resistance to imatinib and all second-generation TKIs [113]. As far as ponatinib is concerned, recent in vitro data suggest that individual CMs have differential responses to ponatinib, ranging from sensitive to highly resistant [114]. Interestingly, CMs including the T315I or F317L revealed a particularly high resistance to ponatinib, whereas several other CMs conferred an intermediate level of resistance which could be overcome by employing the appropriate dose of the kinase inhibitor [114]. This consideration may be of importance in view of the current tendency to reduce the dose of ponatinib in order to prevent the occurrence of severe side effects. The awareness that certain CMs could be suppressed or eliminated by using an adequate ponatinib dosing scheme can be of clinical relevance in specific situations. The detection of specific mutations (or mutation combinations) may therefore not only influence TKI selection, but may also guide the dosing regimen in certain instances. Screening for BCR-ABL1 KD mutations is recommended by the ELN [115] and the NCCN [30] in CML patients who fail to achieve the established milestones of molecular response (EMR, MMR) or who lose these response levels during therapy as well as in patients who present in or progress to AP and BC. In Ph+ ALL, the NCCN [116] and the ESMO [117] recommend BCR-ABL1 mutation screening in relapsed/refractory cases, although the relatively common occurrence of mutations already at the time of diagnosis would argue in favor of early implementation of mutation screening. Recent data indicate that the presence of low-level mutations below the detection limit of Sanger sequencing, but amenable to detection by more sensitive techniques such as next-generation sequencing (NGS), can be of prognostic relevance [118, 119]. Some authors even argue that patients should be screened for low-level mutations at regular intervals until the achievement of MMR in order to provide a basis for timely clinical intervention [119]. The recent introduction of NGS into routine diagnostics is therefore challenging the role of Sanger sequencing as the gold standard for BCR-ABL1 KD mutation screening.

### BCR-ABL1 mutation status and TKI selection: how to critically use “heatmaps”

Besides CMs, clinical decision making may be challenging also for several individual mutations. Indeed, for individual mutations in the BCR-ABL1 KD, specific recommendations for the appropriate TKI choice are provided by the NCCN and ELN, based on in vitro data and clinical experience. However, the available recommendations are restricted to a limited spectrum of commonly occurring mutations (Table 5). In the presence of mutations not covered by the indicated recommendations, published heatmaps indicating the responsiveness of specific mutations to various TKIs are routinely used by physicians for selecting the most adequate treatment approach, in addition to considerations based on individual co-morbidities and other risk factors. Currently available heatmaps highlight the expected responsiveness of mutant subclones to individual TKIs by a traffic light color code, where green indicates sensitivity, red resistance, and yellow an intermediate response [113, 120–122]. However, the indications provided by individual heatmaps must be interpreted with great caution. It is necessary to bear in mind that the heatmaps are based on data generated in vitro by using cell lines, generally of murine origin, that carry BCR-ABL1 constructs with individual mutations, and the indicated results of TKI sensitivity may not necessarily predict the response in vivo.

**Table 5:** BCR-ABL1 KD mutations that influence the selection of second- or third-generation TKIs

T315I	Ponatinib
F317L/V/I/C, T315A	Nilotinib, bosutinib* (or ponatinib if the patient failed or was unable to tolerate first and second-generation TKIs)
V299L	Nilotinib (or ponatinib if the patient failed or was unable to tolerate first and second-generation TKIs)
Y253H, E255V/K, F359V/I/C	Dasatinib, bosutinib* (or ponatinib if the patient failed or was unable to tolerate first and second-generation TKIs)

*There is very limited data available on mutations associated with clinical resistance to bosutinib in vivo. Some in vitro data suggested that the E255K and, to a lesser extent, the E255V, might be poorly sensitive to bosutinib [120]

There are important differences between the concepts underlying the available heatmaps: some indicate the TKI resistance of mutations only in relation to cells carrying unmutated BCR-ABL1 constructs, without considering the clinically achievable TKI plasma levels, while others show the nanomolar inhibitory concentrations (IC_50_ values) of individual TKIs required for specific mutations. Such differences might explain the fact that the data provided by different heatmaps do not overlap in all instances. In fact, a direct comparison of the predicted TKI responses for individual mutations may reveal major differences between various heatmaps [123]. Moreover, in some instances, detection of a BCR-ABL1 KD mutation in a patient may merely identify a specific leukemic subclone in which TKI resistance is not driven by the KD mutation detected, but potentially by other unidentified genetic changes in the affected cells. In such cases, the BCR-ABL1 KD mutation may only serve as a molecular marker for a resistant subclone, but the heatmap would not reflect the actual responsiveness to individual TKIs.

Based on the considerations outlined above, it can be stated that heatmaps may be used for orientation to support the selection of a TKI expected to show efficacy against a specific mutant subclone. However, the molecular response in vivo should be monitored to assess the biological behavior of the respective subclone. Monitoring can be performed by technical approaches permitting quantitative surveillance of the size of mutant subclones during the course of the disease. Currently, the most common approach to this task is the employment of NGS-based assays [124–126], which can provide a basis for timely modification of treatment, if pertinent.

## Conclusions

Therapeutic advances and technological evolution have significantly improved the way we treat CML and Ph+ ALL patients, monitor response, and counteract resistance. Personalized approaches based on risk, treatment endpoints, and BCR-ABL1 mutation status are becoming reality. Nevertheless, there is still much to be done. In CML, clinical investigation is now focusing on how to best identify, based on the entity of BCR-ABL1 transcript reduction, patients who would really benefit from an early switch, how to increase TFR rates, and how to best select TFR candidates. In Ph+ ALL, clinical studies are investigating how to optimize the use of the currently available treatment options (TKIs, monoclonal antibodies, chemotherapy, transplantation) in an attempt to minimize toxicity and treatment-related mortality while maximizing (molecular) response rates. If the past decade has witnessed the TKI revolution, the next will welcome a fine-tuning of TKI use, with the definition of rational decision algorithms taking into account biological and clinical prognostic/predictive factors, both at diagnosis and dynamically during the course of treatment.

## Abbreviations

ALL: Acute lymphoblastic leukemia
AP: Accelerated phase
BC: Blast crisis
CHR: Complete hematologic response
CM: Compound mutation
CML: Chronic myeloid leukemia
CP: Chronic phase
DFS: Disease-free survival
DMR: Deep molecular response
EBMT: European Society for Blood and Marrow Transplantation
ELN: European Leukemia Net
EMR: Early molecular response
ESMO: European Society of Medical Oncology
EWALL: European Working Group on Adult ALL
GIMEMA: Gruppo Italiano Malattie Ematologiche dell’Adulto
GMALL: German Multicenter Study Group for Adult ALL
IS: International scale
KD: Kinase domain
MMR: Major molecular response
MR: Molecular response
MRD: Minimal residual disease
NCCN: National Comprehensive Cancer Network
NGS: Next-generation sequencing
NILG: Northern Italy Leukemia Group
OS: Overall survival
Ph+: Philadelphia chromosome-positive
RIC: Reduced-intensity conditioning
RQ-PCR: Real-time quantitative reverse transcription-polymerase chain reaction
SCT: Stem cell transplantation
TFR: Treatment-free remission
TKI: Tyrosine kinase inhibitor
TRM: Transplant-related mortality
